# Real-time two-axis control of a spin qubit

**DOI:** 10.1038/s41467-024-45857-0

**Published:** 2024-02-23

**Authors:** Fabrizio Berritta, Torbjørn Rasmussen, Jan A. Krzywda, Joost van der Heijden, Federico Fedele, Saeed Fallahi, Geoffrey C. Gardner, Michael J. Manfra, Evert van Nieuwenburg, Jeroen Danon, Anasua Chatterjee, Ferdinand Kuemmeth

**Affiliations:** 1 Center for Quantum Devices, Niels Bohr Institute, University of Copenhagen, 2100 Copenhagen, Denmark; 2 Lorentz Institute and Leiden Institute of Advanced Computer Science, Leiden University, P.O. Box 9506, 2300 RA Leiden, The Netherlands; 3 QDevil, Quantum Machines, 2750 Ballerup, Denmark; 4 Department of Physics and Astronomy, Purdue University, West Lafayette, IN 47907 USA; 5 Birck Nanotechnology Center, Purdue University, West Lafayette, IN 47907 USA; 6 Elmore Family School of Electrical and Computer Engineering, Purdue University, West Lafayette, IN 47907 USA; 7 School of Materials Engineering, Purdue University, West Lafayette, IN 47907 USA; 8 Department of Physics, Norwegian University of Science and Technology, NO-7491 Trondheim, Norway

**Keywords:** Qubits, Quantum dots

## Abstract

Optimal control of qubits requires the ability to adapt continuously to their ever-changing environment. We demonstrate a real-time control protocol for a two-electron singlet-triplet qubit with two fluctuating Hamiltonian parameters. Our approach leverages single-shot readout classification and dynamic waveform generation, allowing full Hamiltonian estimation to dynamically stabilize and optimize the qubit performance. Powered by a field-programmable gate array (FPGA), the quantum control electronics estimates the Overhauser field gradient between the two electrons in real time, enabling controlled Overhauser-driven spin rotations and thus bypassing the need for micromagnets or nuclear polarization protocols. It also estimates the exchange interaction between the two electrons and adjusts their detuning, resulting in extended coherence of Hadamard rotations when correcting for fluctuations of both qubit axes. Our study highlights the role of feedback in enhancing the performance and stability of quantum devices affected by quasistatic noise.

## Abstract (web summary)

Real-time adaptive control of a qubit has been demonstrated but limited to single-axis Hamiltonian estimation. Here the authors implement two-axis control of a singlet-triplet spin qubit with two fluctuating Hamiltonian parameters, resulting in improved quality of coherent oscillations.

## Introduction

Feedback is essential for stabilizing quantum devices and improving their performance. Real-time monitoring and control of quantum systems allows for precise manipulation of their quantum states^1,2^. In this way, it can help mitigate the effects of quantum decoherence and extend the lifetime of quantum systems for quantum computing and quantum sensing applications^3^, for example in superconducting qubits^4–8^, spins in diamond^9–14^, trapped atoms^15,16^, and other platforms^17–22^.

Among the various quantum-information processing platforms, semiconductor spin qubits^23,24^ are promising for quantum computing because of their long coherence times^25^ and foundry compatibility^26^. Focusing on spin qubits hosted in gate-controlled quantum dots (QDs), two-qubit gate fidelities of 99.5% and single-qubit gate fidelities of 99.8% have recently been achieved in silicon^27^. In germanium, a four-qubit quantum processor based on hole spins enabled all-electric qubit logic and the generation of a four-qubit Greenberger-Horne-Zeilinger state^28^. In gallium arsenide, simultaneous coherent exchange rotations and four-qubit measurements in a 2 × 2 array of singlet-triplets were demonstrated without feedback, revealing site-specific fluctuations of nuclear spin polarizations^29^. In silicon, a six-qubit processor was operated with high fidelities enabling universal operation, reliable state preparation and measurement^30^.

Achieving precise control of gated qubits can be challenging due to their sensitivity to environmental fluctuations, making feedback necessary to stabilize and optimize their performance. Because feedback-based corrections must be performed within the correlation time of the relevant fluctuations, real-time control is essential. Continuous feedback then allows to calibrate the qubit environment and to tune the qubit in real time to maintain high-fidelity gates and improved coherence, for instance by suppressing low-frequency noise and improving *π*-flip gate fidelity^31^. An active reset of a silicon spin qubit using feedback control was demonstrated based on quantum non-demolition readout^32^. Real-time operation of a charge sensor in a feedback loop^33^ maintained the sensor sensitivity for fast charge sensing in a Si/SiGe double quantum dot, compensating for disturbances due to gate-voltage variation and 1/*f* charge fluctuations. A quantum state with higher confidence than what is achievable through traditional thermal methods was initialized by real-time monitoring and negative-result measurements^34^.

This study implements real-time two-axis control of a qubit with two fluctuating Hamiltonian parameters that couple to the qubit along different directions on its Bloch sphere. The protocol involves two key steps: first, rapid estimation of the instantaneous magnitude of one of the fluctuating fields (nuclear field gradient) effectively creates one qubit control axis. This control axis is then exploited to probe in real time the qubit frequency (Heisenberg exchange coupling) across different operating points (detuning voltages). Our procedure allows for counteracting fluctuations along both axes, resulting in an improved quality factor of coherent qubit rotations.

Our protocol integrates a singlet-triplet (ST_0_) spin qubit implemented in a gallium arsenide double quantum dot (DQD)^29^ with Bayesian Hamiltonian estimation^35–39^. Specifically, an FPGA-powered quantum orchestration platform (OPX^40^) repeatedly separates singlet-correlated electron pairs using voltage pulses and performs single-shot readout classifications to estimate on-the-fly the fluctuating nuclear field gradient within the double dot^41^. Knowledge of the field gradient in turn enables the OPX to coherently rotate the qubit between S and T_0_ by arbitrary, user-defined target angles. Differently from previous works, we let the gradient freely fluctuate, without pumping the nuclear field^42^, and instead program the OPX to adjust the baseband control pulses accordingly.

An adaptive second-axis estimation is performed to also probe the exchange interaction between the two electrons. This exchange interaction estimation scheme is not simply an independent repetition of the single-axis estimation protocol^35–39^: the design of the exchange-based free induction decay (FID) pulse sequence depends on the outcome of the first-axis estimation and needs to be computed on the fly. Finally, fluctuations along both axes are measured and corrected, enabling the stable coherent rotation of the qubit around a symmetric axis, essential for performing the Hadamard gate.

Our work introduces a versatile method for enhancing coherent control and stability of spin qubits by harnessing low-frequency environmental fluctuations coupling to the system. As such, it is not limited to the operation of ST_0_ qubits in GaAs. Our implementation of real-time reaction to fluctuating Hamiltonian parameters can find application in other materials and qubit encodings, as it is not necessarily limited to nuclear noise.

## Results

### Device and Bayesian estimation

**Fig. 1:**
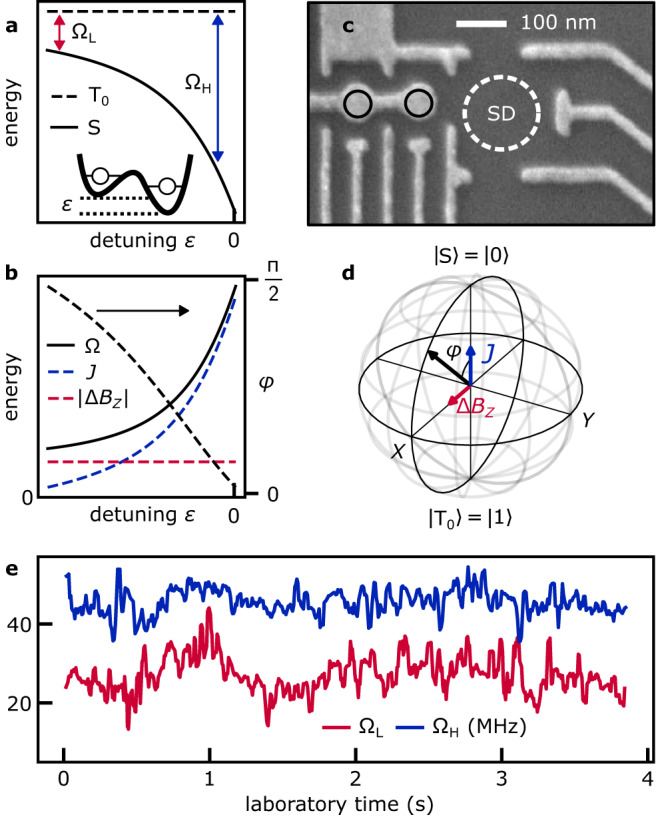
A singlet-triplet (ST_0_) qubit with two fluctuating control axes. **a** The dots' electrical detuning *ε* tunes from a regime of low qubit frequency, Ω_L_, to a regime of high frequency, Ω_H_. States outside the computational space are not plotted. **b** In the first (second) regime, the Overhauser gradient ∣Δ*B*_*z*_∣ (the exchange coupling *J*) dominates the qubit frequency Ω_L_ (Ω_H_) and the polar angle *φ* of the qubit rotation axis. **c** SEM image of the GaAs device^29^, implementing a two-electron double quantum dot (black circles) next to its sensor dot (SD) for qubit readout. **d** *J* and Δ*B*_*z*_ drive rotations of the qubit around two orthogonal axes, providing universal qubit control, as depicted in the Bloch sphere. **e** Uncontrolled fluctuations of the Larmor frequencies Ω_L_ and Ω_H_, estimated in real time on the OPX and plotted with a 30 ms moving average.

We use a top-gated GaAs DQD array^29^ and tune up one of its ST_0_ qubits using the gate electrodes shown in Fig. 1c, at 200 mT in-plane magnetic field in a dilution refrigerator with a mixing-chamber plate below 30 mK. Radio-frequency reflectometry off the sensor dot’s ohmic contact distinguishes the relevant charge configurations of the DQD^43^.

The qubit operates in the (1,1) and (0,2) charge configuration of the DQD. (Integers indicate the number of electrons in the left and right dot.) The electrical detuning *ε* quantifies the difference in the electrochemical potentials of the two dots, which in turn sets the qubit’s spectrum as shown in Fig. 1a. We do not plot the fully spin-polarized triplet states, which are independent of *ε* and detuned in energy by the applied magnetic field. We define *ε* = 0 at the measurement point close to the interdot (1,1)-(0,2) transition, with negative *ε* in the (1,1) region. In the ST_0_ basis, we model the time-dependent Hamiltonian by

1 $${{{{{{{\mathcal{H}}}}}}}}(t)=J(\varepsilon (t))\,\frac{{\sigma }_{z}}{2}+{g}^{*}{\upmu }_{{{{{{{{\rm{B}}}}}}}}}{{\Delta }}{B}_{z}(t)\,\frac{{\sigma }_{x}}{2},$$

which depends on the detuning *ε* that controls the exchange interaction between the two electrons, *J*(*ε*(*t*)), and the component of the Overhauser gradient parallel to the applied magnetic field between the two dots, Δ*B*_*z*_(*t*). *σ*_*i*_ are the Pauli operators, g^*^ is the effective g-factor, and μ_B_ is the Bohr magneton. In the following, we drop the time dependence of the Hamiltonian parameters for ease of notation. On the Bloch sphere of the qubit (Fig. 1d), eigenstates of the exchange interaction, $\left\vert {{{{{{{\rm{S}}}}}}}}\right\rangle$ and $\left\vert {{{{{{{{\rm{T}}}}}}}}}_{0}\right\rangle$, are oriented along *Z*, while Δ*B*_*z*_ enables rotations along *X*.

The qubit is manipulated by voltage pulses applied to the plunger gates of the DQD, and measured near the interdot (1,1)-(0,2) transition by projecting the unknown spin state of (1,1) onto either the (1,1) charge state ($\left\vert {{{{{{{{\rm{T}}}}}}}}}_{0}\right\rangle$) or the (0,2) charge state ($\left\vert {{{{{{{\rm{S}}}}}}}}\right\rangle$). Each single-shot readout of the DQD charge configuration involves generation, demodulation, and thresholding of a few-microsecond-long radio-frequency burst on the OPX (see Supplementary Fig. 1).

The OPX allows for real-time calculation of the qubit Larmor frequency ${{\Omega }}(\varepsilon )=\scriptstyle\sqrt{{{\Delta }}{B}_{z}^{2}+J{(\varepsilon )}^{2}}$ at different detunings, based on real-time estimates of Δ*B*_*z*_ and *J*(*ε*).

Inspecting the exchange coupling in a simplified Fermi-Hubbard hopping model^23^ and inserting *J*(*ε*) into Eq. (1) suggests two physically distinct regimes [Fig. 1b]: At low detuning, in the (1,1) charge state configuration, the Overhauser gradient dominates the qubit dynamics. In this regime, the qubit frequency reads ${{{\Omega }}}_{{{{{{{{\rm{L}}}}}}}}}\equiv \scriptstyle\sqrt{{{\Delta }}{B}_{z}^{2}+{J}_{{{{{{{{\rm{res}}}}}}}}}^{2}}$, where we have added a small phenomenological term ${J}_{{{{{{{{\rm{res}}}}}}}}}$ to account for a constant residual exchange between the two electrons at low detuning. Such a term may become relevant when precise knowledge of Δ*B*_*z*_ is required, for example for the Hadamard protocol at the end of this study. At high detuning, close to the (1,1)–(0,2) interdot charge transition, exchange interaction between the two electrons dominates, and the qubit frequency becomes ${{{\Omega }}}_{{{{{{{{\rm{H}}}}}}}}}(\varepsilon )\equiv \scriptstyle\sqrt{{{\Delta }}{B}_{z}^{2}+J{(\varepsilon )}^{2}}$. As shown in Fig. 1b, the detuning affects both the Larmor frequency Ω and the polar angle *φ* of the qubit rotation axis $\hat{{{{{{{{\boldsymbol{\omega }}}}}}}}}$, with *φ* approaching 0 in the limit *J*(*ε*) ≫ Δ*B*_*z*_ and *π*/2 if *J*(*ε*) ≪ Δ*B*_*z*_.

Without the possibility of turning off either *J* or Δ*B*_*z*_, the rotation axes of the singlet-triplet qubit are tilted, meaning that pure *X*- and *Z*-rotations are unavailable. In their absence, the estimation of the qubit frequency at different operating points is crucial for navigating the whole Bloch sphere of the qubit. Figure 1e tracks Larmor frequencies Ω_H_ and Ω_L_, both fluctuating over tens of MHz over a period of several seconds, using a real-time protocol as explained later. The presence of low-frequency variations in time traces of Ω_H_ and Ω_L_ suggests that qubit coherence can be extended by monitoring these uncontrolled fluctuations in real time and appropriately compensating qubit manipulation pulses on-the-fly.

To estimate the frequency of the fluctuating Hamiltonian parameters on the OPX, we employ a Bayesian estimation approach based on a series of free-induction-decay experiments^35^. Using *m*_*i*_ to represent the outcome ($| {{{{{{{\rm{S}}}}}}}}\rangle$ or $| {{{{{{{{\rm{T}}}}}}}}}_{0}\rangle$) of the *i*-th measurement after an evolution time *t*_*i*_, the conditional probability *P*(*m*_*i*_∣Ω) is defined as the probability of obtaining *m*_*i*_ given a value of Ω:

2 $$P\left({m}_{i}| {{\Omega }}\right)=\frac{1}{2}\left[1+{r}_{i}\left(\alpha+\beta \cos \left(2\pi {{\Omega }}{t}_{i}\right)\right)\right],$$

where *r*_*i*_ takes a value of 1 ( − 1) if ${m}_{i}=\vert {{{{{{{\rm{S}}}}}}}}\rangle$ ($\left\vert {{{{{{{{\rm{T}}}}}}}}}_{0}\right\rangle$), and *α* and *β* are determined based on the measurement error and axis of rotation on the Bloch sphere.

Applying Bayes’ rule to estimate Ω based on the observed measurements *m*_*N*_, …*m*_1_, which are assumed to be independent of each other, yields the posterior probability distribution $P\left({{\Omega }}\,| {m}_{N},\ldots {m}_{1}\right)$ in terms of a prior uniform distribution ${P}_{0}\left({{\Omega }}\right)$ and a normalization constant ${{{{{{{\mathcal{N}}}}}}}}$:

3 $$P\left({{\Omega }}\,| {m}_{N},\ldots {m}_{1}\right)=	 {P}_{0}\left({{\Omega }}\right){{{{{{{\mathcal{N}}}}}}}}\\ 	 \times \mathop{\prod }\limits_{i=1}^{N}\left[1+{r}_{i}\left(\alpha+\beta \cos \left(2\pi {{\Omega }}{t}_{i}\right)\right)\right].$$

Based on previous works^35,38,39^, we fix *α* = 0.25 and *β* = ±0.5, with the latter value positive when estimating Ω_L_ and negative when estimating Ω_H_. The expectation value 〈Ω〉, calculated over the posterior distribution after all *N* measurements, is then taken as the final estimate of Ω.

### Controlled Overhauser gradient driven rotations

We first implement qubit control using one randomly fluctuating Hamiltonian parameter, through rapid Bayesian estimation of Ω_L_ and demonstration of controlled rotations of a ST_0_ qubit driven by the prevailing Overhauser gradient. Notably, this allows coherent control without a micromagnet^44,45^ or nuclear spin pumping^42^.

**Fig. 2:**
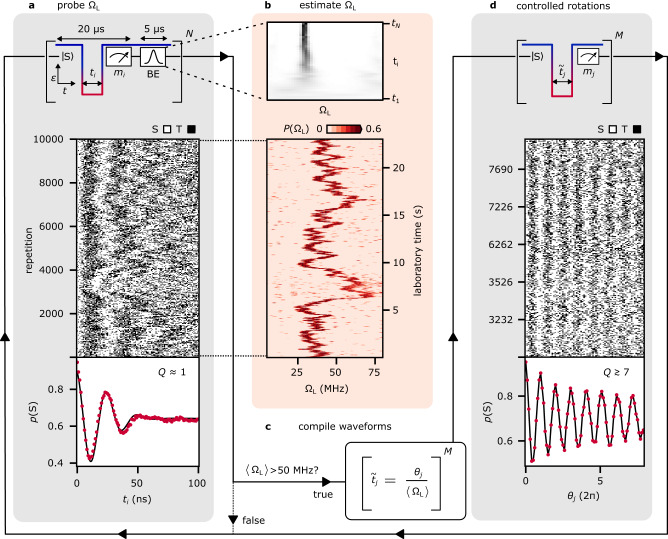
Controlled Overhauser gradient driven rotations of a ST_0_ qubit by real-time Bayesian estimation. One loop (solid arrows) represents one repetition of the protocol. **a** For each repetition, the OPX estimates Ω_L_ by separating a singlet pair for *N* linearly spaced probe times *t*_*i*_ and updating the Bayesian estimate (BE) distribution after each measurement, as shown in the inset of b for one representative repetition. For illustrative purposes, each single-shot measurements *m*_*i*_ is plotted as a white/black pixel, here for *N* = 101 Ω_L_ probe cycles, and the fraction of singlet outcomes in each column is shown as a red dot. **b** Probability distribution *P*(Ω_L_) after completion of each repetition in a. Extraction of the expected value 〈Ω_L_〉 from each row completes Ω_L_ estimation. **c** For each repetition, unless 〈Ω_L_〉 falls below a user-defined minimum (here 50 MHz), the OPX adjusts the separation times ${\tilde{t}}_{j}$, using its real-time knowledge of 〈Ω_L_〉, to rotate the qubit by user-defined target angles ${\theta }_{j}={\tilde{t}}_{j}\,\langle {{{\Omega }}}_{{{{{{{{\rm{L}}}}}}}}}\rangle$. **d** To illustrate the increased coherence of Overhauser gradient driven rotations, we task the OPX to perform *M* = 80 evenly spaced *θ*_*j*_ rotations. Single-shot measurements *m*_*j*_ are plotted as white/black pixels, and the fraction of singlet outcomes in each column is shown as a red dot.

Ω_L_ is estimated from the pulse sequence shown in Fig. 2a: for each repetition a singlet pair is initialized in (0,2) and subsequently detuned deep in the (1, 1) region (*ε*_L_ ≈ − 40 mV) for *N* = 101 linearly spaced separation times *t*_*i*_ up to 100 ns. After each separation, the qubit state, $\left\vert {{{{{{{\rm{S}}}}}}}}\right\rangle$ or $\left\vert {{{{{{{{\rm{T}}}}}}}}}_{0}\right\rangle$, is assigned by thresholding the demodulated reflectometry signal *V*_rf_ near the (1,1)-(0,2) interdot transition and updating the Bayesian probability distribution of Ω_L_ according to the outcome of the measurement. After measurement *m*_*N*_, the initially uniform distribution has narrowed [inset of Fig. 2b, with white and black indicating low and high probability], allowing the extraction of 〈Ω_L_〉 as the estimate for Ω_L_. For illustrative purposes, we plot in Fig. 2a the *N* single-shot measurements *m*_*i*_ for 10,000 repetitions of this protocol, which span a period of about 20 s, and in Fig. 2b the associated probability distribution *P*(Ω_L_) of each repetition. The quality of the estimation seems to be lower around a laboratory time of 6 seconds, coinciding with a reduced visibility of the oscillations in panel 2a. We attribute this to an enhanced relaxation of the triplet state during readout due to the relatively high ∣Δ*B*_*z*_∣ gradient during those repetitions^46^. The visibility could be improved by a latched or shelved read-out^47,48^ or energy-selective tunneling-based readout^38^.

Even though the rotation speed around ${\hat{{{{{{{{\boldsymbol{\omega }}}}}}}}}}_{{{{{{{{\rm{L}}}}}}}}}$ at low detuning is randomly fluctuating in time, knowledge of 〈Ω_L_〉 allows controlled rotations by user-defined target angles. To show this, we task the OPX in Fig. 2d to adjust the separation times ${\tilde{t}}_{j}$ in the pulse sequence to rotate the qubit by *M* = 80 different angles ${\theta }_{j}={\tilde{t}}_{j}\,\langle {{{\Omega }}}_{{{{{{{{\rm{L}}}}}}}}}\rangle$ between 0 and 8*π*. In our notation, the tilde in a symbol $\tilde{x}$ indicates that the waveform parameter *x* is computed dynamically on the OPX. To reduce the FPGA memory required for preparing waveforms with nanosecond resolution, we perform controlled rotations only if the expected Ω_L_ is larger than an arbitrarily chosen minimum of 50 MHz. (The associated IF statement and waveform compilation then takes about 40 μs on the FPGA.) This reduces the number of precomputed waveforms needed for the execution of pulses with nanosecond-scale granularity, for which we use the OPX baked waveforms capability. Accordingly, the number of rows in Fig. 2d (1450) is smaller than in panel a, and we only label a few selected rows with their repetition number.

To show the increased rotation-angle coherence of controlled ∣Δ*B*_*z*_∣-driven rotations, we plot the average of all 1450 repetitions of Fig. 2d and compare the associated quality factor, *Q* ≳ 7, with that of uncontrolled oscillations, *Q* ~ 1 (we define the quality factor as the number of oscillations until the amplitude is 1/e of its original value). The average of the uncontrolled S-T_0_ oscillations in Fig. 2a can be fit by a decay with Gaussian envelope (solid line), yielding an inhomogeneous dephasing time ${T}_{2}^{*}\,\approx\, 30\,{{{{{{{\rm{ns}}}}}}}}$ typical for ST_0_ qubits in GaAs^49^. We associate the relatively smaller amplitude of stabilized qubit oscillations with the low-visibility region around 6 seconds in Fig. 2d, discussed earlier. Excluding such regions by post selection increases the visibility and quality factor of oscillations (see Supplementary Fig. 4). Overall, the results presented in this section exemplify how adaptive baseband control pulses can operate a qubit reliably, out of slowly fluctuating environments.

### Real-time two-axis estimation

In addition to nuclear spin noise, ST_0_ qubits are exposed to electrical noise in their environment, which affects the qubit splitting in particular at higher detunings. It is therefore important to examine and mitigate low-frequency noise at different operating points of the qubit. In the previous section, the qubit frequency Ω_L_ was estimated entirely at low detuning where the Overhauser field gradient dominates over the exchange interaction. In order to probe and stabilize also the second control axis, namely *J*-driven rotations corresponding to small *φ* in Fig. 1d, we probe the qubit frequency Ω_H_ at higher detunings, using a similar protocol with a modified qubit initialization.

**Fig. 3:**
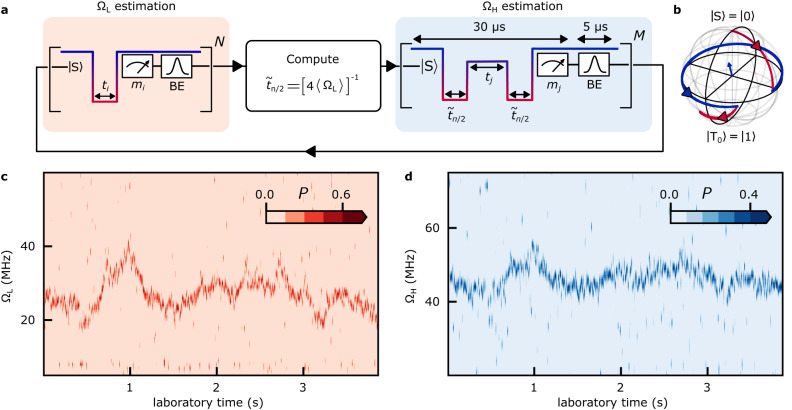
Real-time Bayesian estimation of two control axes. **a** One repetition of the two-axis estimation protocol. After estimating Ω_L_ from *N* = 101 *t*_*i*_ probe cycles (Fig. 2a), the OPX computes on-the-fly the pulse duration ${\tilde{t}}_{\pi /2}$ required to initialize the qubit near the equator of the Bloch sphere by a diabatic Ω_L_(*π*/2) pulse. After the Ω_L_(*π*/2) pulse, the qubit evolves for time *t*_*j*_ under exchange interaction before another Ω_L_(*π*/2) pulse initiates readout. After each single-shot measurement *m*_*j*_, the OPX updates the BE distribution of Ω_H_. Similar to *t*_*i*_ in the Ω_L_ estimation, *t*_*j*_ is spaced evenly between 0 and 100 ns across *M* = 101 exchange probe cycles. **b** Qubit evolution on the Bloch sphere during one exchange probe cycle. **c** Each column plots *P*(Ω_L_) after completion of the Ω_L_ estimation in each protocol repetition. **d** Each column plots *P*(Ω_H_) after completion of the Ω_H_ estimation in each protocol repetition.

Free evolution of the initial state $\left\vert {{{{{{{\rm{S}}}}}}}}\right\rangle$ around ${\hat{{{{{{{{\boldsymbol{\omega }}}}}}}}}}_{{{{{{{{\rm{L}}}}}}}}}$ would result in low-visibility exchange-driven oscillations because of the low value of *φ*. To circumvent this problem, we precede the Ω_H_ estimation by one repetition of Ω_L_ estimation, as shown in Fig. 3a. This way, real-time knowledge of 〈Ω_L_〉 allows the initial state $\left\vert {{{{{{{\rm{S}}}}}}}}\right\rangle$ to be rotated to a state near the equator of the Bloch sphere, before it evolves freely for probing Ω_H_. This rotation is implemented by a diabatic detuning pulse from (0,2) to *ε*_L_ (diabatic compared to the interdot tunnel coupling) for time ${\tilde{t}}_{\pi /2}$, corresponding to a rotation of the qubit around ${\hat{{{{{{{{\boldsymbol{\omega }}}}}}}}}}_{{{{{{{{\rm{L}}}}}}}}}$ by an angle ${{{\Omega }}}_{{{{{{{{\rm{L}}}}}}}}}{\tilde{t}}_{\pi /2}=\pi /2$. After evolution for time *t*_*j*_ under finite exchange, another *π*/2 rotation around ${\hat{{{{{{{{\boldsymbol{\omega }}}}}}}}}}_{{{{{{{{\rm{L}}}}}}}}}$ rotates the qubit to achieve a high readout contrast in the ST_0_ basis, as illustrated on the Bloch sphere in Fig. 3b.

As a side note, we mention that in the absence of knowledge of the Overhauser field gradient, the qubit would traditionally be initialized near the equator by adiabatically reducing detuning from (0,2) to the (1,1) charge configuration, and a reverse ramp for readout. Such adiabatic ramps usually last several microseconds each, while our ${\tilde{t}}_{\pi /2}$ pulses typically take less than 10 ns, thereby significantly shortening each probe cycle.

For the estimate of Ω_H_, the Bayesian probability distribution of Ω_H_ is updated after each of the *M* = 101 single-shot measurement *m*_*j*_, each corresponding to a separation time *t*_*j*_ that is evenly stepped from 0 to 100 ns. The Bayesian probability distributions of both Ω_L_ and Ω_H_ are shown in Fig. 3c and d, respectively, with the latter being conditioned on 20 MHz < 〈Ω_L_〉 < 40 MHz to reduce the required FPGA memory.

This section demonstrated a real-time baseband control protocol that enables manipulation of a spin qubit on the entire Bloch sphere.

### Controlled exchange-driven rotations

**Fig. 4:**
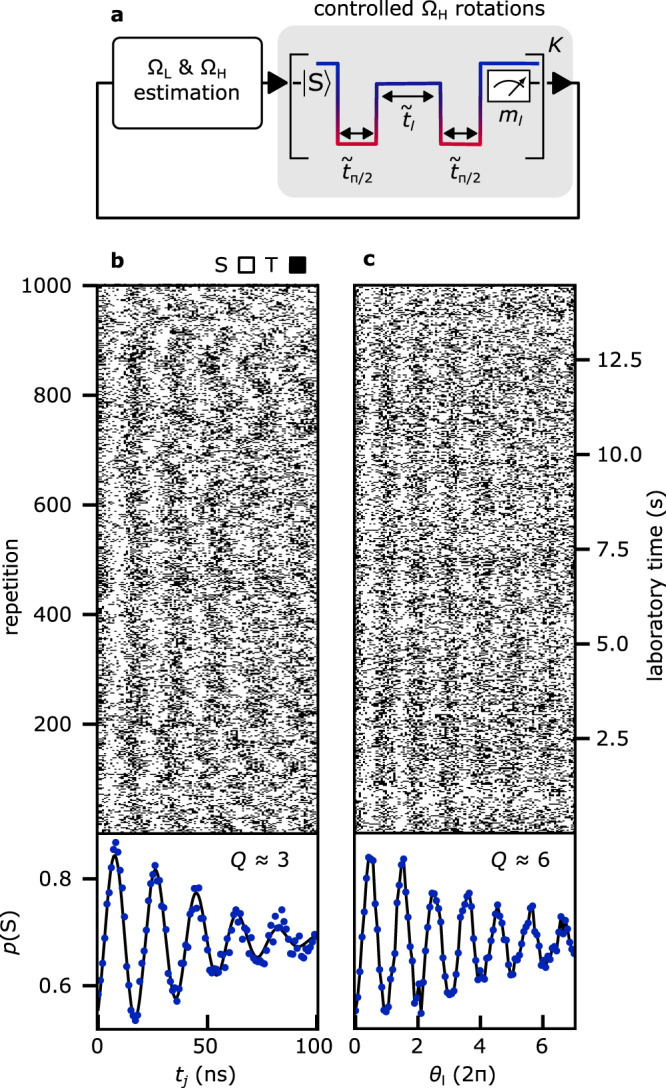
Real-time-controlled exchange-driven qubit rotations. **a** One repetition of the exchange rotation protocol. After estimation of Ω_L_ and Ω_H_ as in Fig. 3, the OPX adjusts exchange duration times ${\tilde{t}}_{l}$, using real-time knowledge of 〈Ω_H_〉, to rotate the qubit by user-defined target angles ${\theta }_{l}={\tilde{t}}_{l}\,\langle {{{\Omega }}}_{{{{{{{{\rm{H}}}}}}}}}\rangle$. Pulse durations ${\tilde{t}}_{\pi /2}$ for qubit initialization and readout use real-time knowledge of 〈Ω_L_〉. **b** Each row plots measurements *m*_*j*_ from one protocol repetition, here *M* = 101 exchange probe outcomes. **c** Each row plots measurement *m*_*l*_ from one protocol repetition, here *K* = 101 controlled-exchange-rotation outcomes. To illustrate the increased coherence of controlled exchange rotations, we also plot in b and c the fraction of singlet outcomes of each column.

Using Bayesian inference to estimate control axes in real-time offers new possibilities for studying and mitigating qubit noise at all detunings. Figure 4a describes the real-time controlled exchange-driven rotations protocol aimed at stabilizing frequency fluctuations of the qubit at higher detunings. Following the approach of Fig. 3, we first estimate Ω_L_ and Ω_H_ using real-time Bayesian estimation. We then use our knowledge of Ω_H_ to increase the rotation angle coherence of the qubit where the exchange coupling is comparable with the Overhauser field gradient.

As illustrated in Fig. 4a, the qubit control pulses now respond in real time to both qubit frequencies Ω_L_ and Ω_H_. Similar to the previous section, after determining 〈Ω_L_〉 and confirming that 30 MHz < 〈Ω_L_〉 < 50 MHz is fulfilled, the qubit is initialized near the equator of the Bloch sphere by fast diabatic Ω_L_(*π*/2) pulses, followed by an exchange-based FID that probes Ω_H_. Based on the resulting 〈Ω_H_〉, the OPX adjusts the separation times ${\tilde{t}}_{l}$ to rotate the qubit by user-defined target angles ${\theta }_{l}={\tilde{t}}_{l}\,\langle {{{\Omega }}}_{{{{{{{{\rm{H}}}}}}}}}\rangle$.

To show the resulting improvement of coherent exchange oscillations, we plot in Fig. 4c the interleaved *K* = 101 measurements *m*_*l*_ and compare them in Fig. 4b to the *M* = 101 measurements *m*_*j*_. Fitting the average of the uncontrolled rotations by an oscillatory fit with Gaussian envelope decay yields ${T}_{{{{{{{{\rm{el}}}}}}}}}^{*}\,\approx\, 60\,{{{{{{{\rm{ns}}}}}}}}$ and *Q* ≈ 3, presumably limited by electrical noise^49^, while the quality factor of the controlled rotations is enhanced by a factor of two, *Q* ≈ 6.

The online control of exchange-driven rotations using Bayesian inference stabilizes fluctuations of the qubit frequency at higher detunings, where fluctuations are more sensitive to detuning noise. Indeed, we attribute the slightly smaller quality factor, relative to Overhauser-driven rotations in Fig. 2d, to an increased sensitivity to charge noise at larger detuning, which, owing to its high-frequency component, is more likely to fluctuate on the estimation timescales^36^.

This section established for the first time stabilization of two rotation axes of a spin qubit. This advancement should allow for stabilized control over the entire Bloch sphere, which we demonstrate in the next section.

### Hadamard rotations

**Fig. 5:**
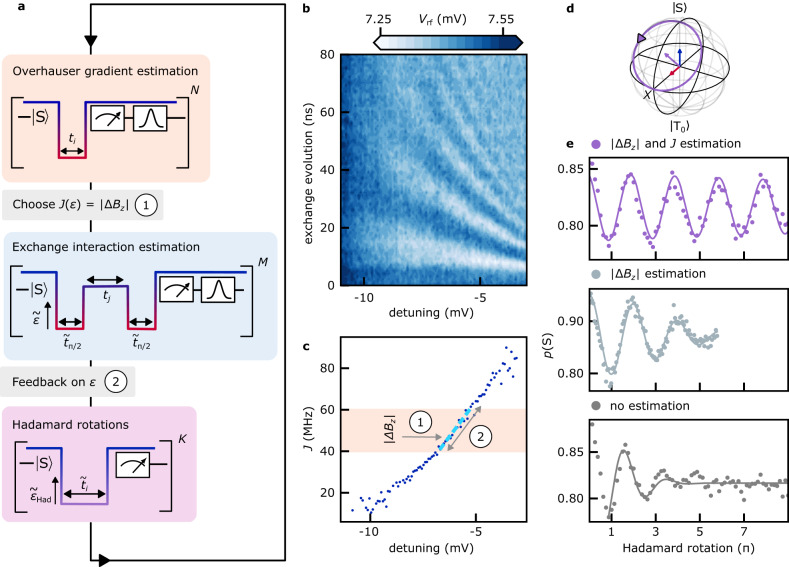
Real-time universal ST_0_ control demonstrated by Hadamard rotations. **a** Hadamard rotation protocol. After estimating Ω_L_, *ε* is chosen in real-time such that *J*(*ε*) = ∣Δ*B*_*z*_∣, based on a linearized offline model from panel c. If $40\,{{{{{{{\rm{MHz}}}}}}}}\, < \,\left\langle | {{\Delta }}{B}_{z}| \right\rangle \, < \, 60\,{{{{{{{\rm{MHz}}}}}}}}$, the detuning is adjusted to account for deviations of the prevailing *J* from the offline model. Real-time knowledge of ${{{\Omega }}}_{{{{{{{{\rm{Had}}}}}}}}}=\sqrt{2}\,| {{\Delta }}{B}_{z}|$ then dictates ${\tilde{t}}_{i}$ to achieve a user-defined Hadamard rotation angle. **b** Averaged exchange driven FID as a function of detuning and evolution time. Here, a diabatic Ω_L_(*π*/2) pulse initializes the qubit near the equator of the Bloch sphere, prior to free exchange evolution, and subsequently prepares it for readout. **c** *J* as a function of *ε* extracted offline from b, as well as a linearized model (dashed line) used in the two feedback steps of panel a. **d** Hadamard rotation depicted on the Bloch sphere. **e** Measurement of Hadamard rotations with ∣Δ*B*_*z*_∣ and *J* estimation (purple, top panel), only ∣Δ*B*_*z*_∣ estimation (light gray, middle panel), and without the feedback shown in a (dark gray, bottom panel).

In this experiment, we demonstrate universal ST_0_ control that corrects for fluctuations in all Hamiltonian parameters. We execute controlled Hadamard rotations around ${\hat{{{{{{{{\boldsymbol{\omega }}}}}}}}}}_{{{{{{{{\rm{Had}}}}}}}}}$, as depicted by the trajectory on the Bloch sphere of Fig. 5d, by selecting the detuning *ε*_Had_ in real time such that *J*(*ε*_Had_) = ∣Δ*B*_*z*_∣. To achieve this, we do not assume that Δ*B*_*z*_ = Ω_L_ (i.e. we allow contributions of ${J}_{{{{{{{{\rm{res}}}}}}}}}$ to Ω_L_) or that *J* = Ω_L_ (i.e. we allow contributions of Δ*B*_*z*_ to Ω_H_). The full protocol is detailed in Supplementary Discussion.

Real-time knowledge of both Δ*B*_*z*_ and *J* would potentially benefit two-qubit gate fidelities^50^ and the resonant-driving approach of previous works^35,38,39^. In the resonant implementation, constrained to the operating regime ∣Δ*B*_*z*_∣ ≫ *J*, low-frequency fluctuations of *J* result in transverse noise that causes dephasing and phase shifts of the Rabi rotations^51,52^.

In previous sections, we have shown how to probe the qubit Larmor frequencies Ω_H_ and Ω_L_ at different detunings in real time and correct for their fluctuations. Now, we simultaneously counteract fluctuations in *J* and ∣Δ*B*_*z*_∣ on the OPX in order to perform the Hadamard gate. As we do not measure the sign of Δ*B*_*z*_, we identify the polar angle of ${\hat{{{{{{{{\boldsymbol{\omega }}}}}}}}}}_{{{{{{{{\rm{Had}}}}}}}}}$ as either *φ* = *π*/4 or − *π*/4. In other words, starting from the singlet state, the qubit rotates towards + *X* on the Bloch sphere for one sign of Δ*B*_*z*_, and towards − *X* for the other sign. The sign of the gradient may change over long time scales due to nuclear spin diffusion (on the order of many seconds^41^), but the measurement outcomes of our protocol are expected to be independent of the sign.

The relative sign of Overhauser gradients becomes relevant for multi-qubit experiments^53^, and could be determined^54^ by comparing the relaxation time of the ground state (e.g. $| \uparrow \downarrow\, \rangle$) of Δ*B*_*z*_ with its excited state ($| \downarrow \uparrow \,\rangle$). Such diagnostic sign-probing cycles on the FPGA should not require more than a few milliseconds, negligible compared to the expected time between sign reversals.

In preparation for our protocol, we first extract the time-averaged exchange profile by performing exchange oscillations as a function of evolution time [Fig. 5b]. Removing contributions of Δ*B*_*z*_ to Ω_H_ then yields *J*(*ε*) in Fig. 5c. A linear approximation in the target range 40 MHz < 〈 *J*〉 < 60 MHz (dashed blue line) is needed later on the OPX to allow initial detuning guesses when tuning up *J*(*ε*) = ∣Δ*B*_*z*_∣. We also provide the OPX with a value for the residual exchange at low detuning, ${J}_{{{{{{{{\rm{res}}}}}}}}}\,\approx \,20\,{{{{{{{\rm{MHz}}}}}}}}$, determined offline as described in Supplementary Fig. 5.

As illustrated in Fig. 5a, the Hadamard rotation protocol starts by estimating ∣Δ*B*_*z*_∣ from Ω_L_, taking into account a constant residual exchange by solving ${{\Delta }}{B}_{z}^{2}={{{\Omega }}}_{{{{{{{{\rm{L}}}}}}}}}^{2}-{J}_{{{{{{{{\rm{res}}}}}}}}}^{2}$.

Next, an initial value of *ε*_Had_ is chosen based on the linear offline model to fulfill *J*(*ε*_Had_) = ∣Δ*B*_*z*_∣ [feedback in panel (a,c)]. To detect any deviations of the prevailing *J* from the offline model, an exchange-driven FID is performed at *ε*_Had_ to estimate *J* from Ω_H_, using ${J}^{2}={{{\Omega }}}_{{{{{{{{\rm{H}}}}}}}}}^{2}-{{\Delta }}{B}_{z}^{2}$.

Any deviation of 〈*J*〉 from the target value ∣Δ*B*_*z*_∣ is subsequently corrected for by updating *ε*_Had_ based on the linearized *J*(*ε*) model [feedback in panels (a,c)]. Matching *J* to ∣Δ*B*_*z*_∣ in the two detuning feedback steps each takes about 400 ns on the OPX. Finally, real-time knowledge of ${{{\Omega }}}_{{{{{{{{\rm{Had}}}}}}}}}\equiv \sqrt{2}| {{\Delta }}{B}_{z}|$ is used to generate the free evolution times ${\tilde{t}}_{i}$, spent at the updated value ${\tilde{\varepsilon }}_{{{{{{{{\rm{Had}}}}}}}}}$, in order to perform Hadamard rotations by *K* user defined target angles.

The resulting Hadamard oscillations are shown in Fig. 5e (top panel) and fitted with an exponentially decaying sinusoid, indicating a quality factor *Q* > 5. (According to this naive fit, the amplitude drops to 1/e over approximately 40 rotations, although we have not experimentally explored rotation angles beyond 9*π*.) In comparison to exchange-controlled rotations from Fig. 4c, the Hadamard rotations are more stable, which we attribute to the additional feedback on detuning that fixes the oscillation axis and decreases sensitivity to charge noise.

To illustrate the crucial role of real-time estimation for this experiment, we also performed rotation experiments that do not involve any real-time estimation and feedback cycles (dark gray data, bottom panel), as follows. Within minutes after performing the controlled Hadamard rotations (purple data), we executed Hadamard rotations assuming a fixed value of $| {{\Delta }}{B}_{z}|=\overline{| {{\Delta }}{B}_{z}| }$, i.e. by pulsing to a fixed detuning value corresponding to $J({\varepsilon }_{{{{{{{{\rm{H}}}}}}}}})=\overline{| {{\Delta }}{B}_{z}| }$ according to the offline model. Here, $\overline{| {{\Delta }}{B}_{z}| }\approx 40$ MHz is the average Overhauser gradient that we observed just before executing the Hadamard protocol. Not surprisingly, the quality factor of the resulting Hadamard-like oscillations is low and the rotation angle deviates from the intended target angle, likely due to the Overhauser gradient having drifted in time. As a side note, we mention that the purple data in Fig. 5e constitutes an average over 5000 repetitions, corresponding to a total acquisition time of 2 minutes including Overhauser and exchange estimation cycles. In contrast, the dark gray data also constitutes an average over 5000 repetitions, but only required 15 seconds because of the omission of all estimation and feedback cycles.

To verify that the enhancement in *Q* is not solely due to the more accurate knowledge of ∣Δ*B*_*z*_∣, we also performed Hadamard rotations only using the estimation of ∣Δ*B*_*z*_∣. The FPGA was programmed to perform a measurement where the initialized singlet is pulsed to a fixed detuning *J*(*ε*_H_) ≈ 20 MHz to perform a Hadamard rotation, only if the estimated ∣Δ*B*_*z*_∣ on the FPGA satisfies $17\,{{{{{{{\rm{MHz}}}}}}}}\, < \,\left\langle | {{\Delta }}{B}_{z}| \right\rangle \, < \,23\,{{{{{{{\rm{MHz}}}}}}}}$. We then post select the repetitions where $19.5\,{{{{{{{\rm{MHz}}}}}}}}\, < \,\left\langle | {{\Delta }}{B}_{z}| \right\rangle \, < \, 20.5\,{{{{{{{\rm{MHz}}}}}}}}$. Fitting this by an oscillatory fit with Gaussian envelope decay yields ${T}_{{{{{{{{\rm{el}}}}}}}}}^{*}\,\approx\, 70\,{{{{{{{\rm{ns}}}}}}}}$, *Q* ≈ 2.0 and frequency ≈ 29 MHz.

In Fig. 5e we compare these data (light gray, middle panel) with the cases where the FPGA estimated both ∣Δ*B*_*z*_∣ and *J* (purple, top panel) and where the microprocessor does not perform any estimation but simply pulses to *J*(*ε*_H_) to perform the rotations (dark gray, bottom panel). (In the middle panel the horizontal axis was rescaled to the Hadamard evolution time using the fitted frequency ≈ 29 MHz.) We see that (i) a reduction of the uncertainty in ∣Δ*B*_*z*_∣ from ≈ 30 MHz (r.m.s.) to ≈ 2 MHz (dark gray to light gray) does not yield a proportional gain in *Q* and (ii) the improvement in *Q* when including estimation of *J* (light gray to purple) is much larger than can be justified solely by the slight further reduction of the uncertainty in ∣Δ*B*_*z*_∣ (roughly from ≈ 2 MHz to ≈ 1 MHz). This demonstrates the crucial contribution of the estimations along both axes in the improvement of our Hadamard gate quality factor.

Further evidence for the fluctuating nature of non-stabilized Hadamard rotations is discussed in Supplementary Fig. 6.

The stabilized Hadamard rotations demonstrate real-time feedback control based on Bayesian estimation of *J* and ∣Δ*B*_*z*_∣, and suggest a significant improvement in coherence for ST_0_ qubit rotations around a tilted control axis. Despite the presence of fluctuations in all Hamiltonian parameters, we report effectively constant amplitude of Hadamard oscillations, with a reduced visibility that we tentatively attribute to estimation and readout errors.

## Discussion

Our experiments demonstrate the effectiveness of feedback control in stabilizing and improving the performance of a singlet-triplet spin qubit. The protocols presented showcase two-axis control of a qubit with two fluctuating Hamiltonian parameters, made possible by implementing online Bayesian estimation and feedback on a low-latency FPGA-powered qubit control system. Real-time estimation allows control pulses to counteract fluctuations in the Overhauser gradient, enabling controlled Overhauser-driven rotations without the need for micromagnets or nuclear polarization protocols. Notably, even in the absence of a deterministic component of the Hamiltonian purely noise-driven coherent rotations of a two-level quantum system were demonstrated.

The approach is extended to the real-time estimation of the second rotation axis, dominated by exchange interaction, which we then combine with an adaptive feedback loop to generate and stabilize Hadamard rotations. In particular, executing the Hadamard gate involves (i) sequentially executing two distinct estimation cycles, where the design of the second cycle relies on the outcomes of the first, (ii) correlating the detected frequencies to distinguish independent fluctuations of the two control axes, and (iii) utilizing this correlated information to dynamically construct and execute a Hadamard gate. These steps demand real-time adaptive estimations and signal generations throughout the protocol, which has not been demonstrated before. A constant Overhauser field gradient, whether stemming from nuclear spin pumping or a micromagnet, is expected to further improve the feedback control. From this perspective, our work represents a worst-case scenario, demonstrating the effectiveness of our experimental technique.

Our protocols assume that Δ*B*_*z*_ does not depend on the precise dot detuning in the (1,1) configuration and remains constant on the time scale of one estimation. Similarly, stabilization of exchange rotations is only effective for electrical fluctuations that are slow compared to one estimation. Therefore, we expect potential for further improvements by more efficient estimation methods, for example through adaptive schemes^55^ for Bayesian estimation from fewer samples, or by taking into account the statistical properties of a time-varying signal described by a Wiener process^56^ or a nuclear spin bath^57^. Machine learning could be used to predict the qubit dynamics^58–60^, possibly via long short-term memory artificial neural networks as reported for superconducting qubits^61^. While our current qubit cycle time (approximately 30 μs) is dominated by readout and qubit initialization, it can potentially be reduced to a few microseconds through faster qubit state classification, such as enhanced latched readout^48^, and faster reset, such as fast exchange of one electron with the reservoir^62^. Our protocol could be modified for real-time non-local noise correlations^63^ or in-situ qubit tomography using fast Bayesian tomography^64^ to study the underlying physics of the noisy environment, thereby providing qualitatively new insights into processes affecting qubit coherence and multi-qubit error correction.

Beyond ST_0_ qubits, our protocols uncover new perspectives on coherent control of quantum systems manipulated by baseband pulses. This work represents a significant advancement in quantum control by implementing an FPGA-powered technique to stabilize in real time the qubit frequency at different manipulation points.

## Methods

### Experimental setup

We use an Oxford Instruments Triton 200 cryofree dilution refrigerator with base temperature below 30 mK. The experimental setup employs a Quantum Machines OPX+ for radio-frequency (RF) reflectometry and gate control pulses. The RF carrier frequency is ≈158 MHz and the gate control pulses sent to the left and right plunger gates of the DQD are filtered with low-pass filters (≈220 MHz) at room temperature, before being attenuated at different stages of the refrigerator. Low-frequency tuning voltages (high-frequency baseband waveforms) are applied by a QDAC^65^ (OPX) via a QBoard high-bandwidth sample holder^66^.

### Measurement details

Before qubit manipulation, an additional reflectometry measurement is taken as a reference to counteract slow drifts in the sensor dot signal. At the end of each qubit cycle, a ≈ 1 μs long pulse is applied to discharge the bias tee. As the qubit cycle period (tens of μs) is much shorter than the bias tee cutoff (≈300 Hz), we do not correct the pulses for the transfer function of the bias tee.

### Supplementary information

Supplementary Information

Peer Review File

## Data Availability

The datasets generated and analyzed during the current study are available from the corresponding authors (F.B., A.C., and F.K.) upon request.

## References

[1] Wiseman HM (1994). Quantum theory of continuous feedback. Phys. Rev. A.

[2] Wiseman, H. M. & Milburn, G. J.*Quantum Measurement and Control* (Cambridge University Press, 2009).

[3] Zhang J, xi Liu Y, Wu R-B, Jacobs K, Nori F (2017). Quantum feedback: Theory, experiments, and applications. Phys. Rep..

[4] Vijay R (2012). Stabilizing Rabi oscillations in a superconducting qubit using quantum feedback. Nature.

[5] Campagne-Ibarcq P (2013). Persistent control of a superconducting qubit by stroboscopic measurement feedback. Phys. Rev. X.

[6] de Lange G (2014). Reversing quantum trajectories with analog feedback. Phys. Rev. Lett..

[7] Masuyama Y (2018). Information-to-work conversion by Maxwell’s demon in a superconducting circuit quantum electrodynamical system. Nat. Commun..

[8] Vepsäläinen A (2022). Improving qubit coherence using closed-loop feedback. Nat. Commun..

[9] Blok MS (2014). Manipulating a qubit through the backaction of sequential partial measurements and real-time feedback. Nat. Phys..

[10] Bonato C (2015). Optimized quantum sensing with a single electron spin using real-time adaptive measurements. Nat. Nanotechnol..

[11] Hirose M, Cappellaro P (2016). Coherent feedback control of a single qubit in diamond. Nature.

[12] Cramer J (2016). Repeated quantum error correction on a continuously encoded qubit by real-time feedback. Nat. Commun..

[13] Wu S-H, Turner E, Wang H (2021). Continuous real-time sensing with a nitrogen-vacancy center via coherent population trapping. Phys. Rev. A.

[14] Turner E, Wu S-H, Li X, Wang H (2022). Spin-based continuous Bayesian magnetic-field estimations aided by feedback control. Phys. Rev. A.

[15] Bushev P (2006). Feedback cooling of a single trapped ion. Phys. Rev. Lett..

[16] Singh K (2023). Mid-circuit correction of correlated phase errors using an array of spectator qubits. Science.

[17] Steck DA, Jacobs K, Mabuchi H, Bhattacharya T, Habib S (2004). Quantum feedback control of atomic motion in an optical cavity. Phys. Rev. Lett..

[18] Sayrin C (2011). Real-time quantum feedback prepares and stabilizes photon number states. Nature.

[19] Wilson DJ (2015). Measurement-based control of a mechanical oscillator at its thermal decoherence rate. Nature.

[20] Rossi M, Mason D, Chen J, Tsaturyan Y, Schliesser A (2018). Measurement-based quantum control of mechanical motion. Nature.

[21] Magrini L (2021). Real-time optimal quantum control of mechanical motion at room temperature. Nature.

[22] Tebbenjohanns F, Mattana ML, Rossi M, Frimmer M, Novotny L (2021). Quantum control of a nanoparticle optically levitated in cryogenic free space. Nature.

[23] Burkard G, Ladd TD, Pan A, Nichol JM, Petta JR (2023). Semiconductor spin qubits. Rev. Mod. Phys..

[24] Chatterjee A (2021). Semiconductor qubits in practice. Nat. Rev. Phys..

[25] Stano P, Loss D (2022). Review of performance metrics of spin qubits in gated semiconducting nanostructures. Nat. Rev. Phys..

[26] Zwerver AMJ (2022). Qubits made by advanced semiconductor manufacturing. Nat. Electron..

[27] Noiri A (2022). Fast universal quantum gate above the fault-tolerance threshold in silicon. Nature.

[28] Hendrickx NW (2021). A four-qubit germanium quantum processor. Nature.

[29] Fedele F (2021). Simultaneous operations in a two-dimensional array of singlet-triplet qubits. PRX Quantum.

[30] Philips SGJ (2022). Universal control of a six-qubit quantum processor in silicon. Nature.

[31] Nakajima T (2020). Coherence of a driven electron spin qubit actively decoupled from quasistatic noise. Phys. Rev. X.

[32] Kobayashi T (2023). Feedback-based active reset of a spin qubit in silicon. npj Quantum Inf..

[33] Nakajima T (2021). Real-time feedback control of charge sensing for quantum dot qubits. Phys. Rev. Appl..

[34] Johnson MAI (2022). Beating the thermal limit of qubit initialization with a Bayesian Maxwell’s demon. Phys. Rev. X.

[35] Shulman MD (2014). Suppressing qubit dephasing using real-time Hamiltonian estimation. Nat. Commun..

[36] Dial OE (2013). Charge noise spectroscopy using coherent exchange oscillations in a singlet-triplet qubit. Phys. Rev. Lett..

[37] Cerfontaine P (2020). Closed-loop control of a GaAs-based singlet-triplet spin qubit with 99.5% gate fidelity and low leakage. Nat. Commun..

[38] Kim J (2022). Approaching ideal visibility in singlet-triplet qubit operations using energy-selective tunneling-based Hamiltonian estimation. Phys. Rev. Lett..

[39] Yun J (2023). Probing two-qubit capacitive interactions beyond bilinear regime using dual Hamiltonian parameter estimations. npj Quantum Inf..

[40] Quantum Machines model OPX+, www.quantum-machines.co

[41] Malinowski FK (2017). Spectrum of the nuclear environment for GaAs spin qubits. Phys. Rev. Lett..

[42] Foletti S, Bluhm H, Mahalu D, Umansky V, Yacoby A (2009). Universal quantum control of two-electron spin quantum bits using dynamic nuclear polarization. Nat. Phys..

[43] Vigneau F (2023). Probing quantum devices with radio-frequency reflectometry. Appl. Phys. Rev..

[44] Wu X (2014). Two-axis control of a singlet–triplet qubit with an integrated micromagnet. Proc. Natl Acad. Sci..

[45] Jang W (2020). Individual two-axis control of three singlet-triplet qubits in a micromagnet integrated quantum dot array. Appl. Phys. Lett..

[46] Barthel C (2012). Relaxation and readout visibility of a singlet-triplet qubit in an Overhauser field gradient. Phys. Rev. B.

[47] Yang CH (2014). Charge state hysteresis in semiconductor quantum dots. Appl. Phys. Lett..

[48] Harvey-Collard P (2018). High-fidelity single-shot readout for a spin qubit via an enhanced latching mechanism. Phys. Rev. X.

[49] Martins F (2016). Noise suppression using symmetric exchange gates in spin qubits. Phys. Rev. Lett..

[50] Petit L (2022). Design and integration of single-qubit rotations and two-qubit gates in silicon above one kelvin. Commun. Mater..

[51] Koppens FHL (2007). Universal phase shift and nonexponential decay of driven single-spin oscillations. Phys. Rev. Lett..

[52] Ramon G, Cywiński Ł (2022). Qubit decoherence under two-axis coupling to low-frequency noises. Phys. Rev. B.

[53] Cerfontaine P, Otten R, Wolfe MA, Bethke P, Bluhm H (2020). High-fidelity gate set for exchange-coupled singlet-triplet qubits. Phys. Rev. B.

[54] Cai X, Connors EJ, Edge LF, Nichol JM (2023). Coherent spin-valley oscillations in silicon. Nat. Phys..

[55] Sergeevich A, Chandran A, Combes J, Bartlett SD, Wiseman HM (2011). Characterization of a qubit Hamiltonian using adaptive measurements in a fixed basis. Phys. Rev. A.

[56] Bonato C, Berry DW (2017). Adaptive tracking of a time-varying field with a quantum sensor. Phys. Rev. A.

[57] Scerri E, Gauger EM, Bonato C (2020). Extending qubit coherence by adaptive quantum environment learning. N. J. Phys..

[58] Mavadia S, Frey V, Sastrawan J, Dona S, Biercuk MJ (2017). Prediction and real-time compensation of qubit decoherence via machine learning. Nat. Commun..

[59] Gupta RS, Biercuk MJ (2018). Machine learning for predictive estimation of qubit dynamics subject to dephasing. Phys. Rev. Appl..

[60] Fiderer LJ, Schuff J, Braun D (2021). Neural-network heuristics for adaptive Bayesian quantum estimation. PRX Quantum.

[61] Koolstra G (2022). Monitoring fast superconducting qubit dynamics using a neural network. Phys. Rev. X.

[62] Botzem T (2018). Tuning methods for semiconductor spin qubits. Phys. Rev. Appl..

[63] Szańkowski P, Trippenbach M, Cywiński Ł (2016). Spectroscopy of cross correlations of environmental noises with two qubits. Phys. Rev. A.

[64] Evans T (2022). Fast Bayesian tomography of a two-qubit gate set in silicon. Phys. Rev. Appl..

[65] QDevil model QDAC-II, www.quantum-machines.co

[66] QDevil model QBoard-I, www.quantum-machines.co

