# Supplementary material for: Real-time two-axis control of a spin qubit
Source: Nat Commun. 2024 Feb 23;15:1676. doi: 10.1038/s41467-024-45857-0 (PMC10891052; doi:10.1038/s41467-024-45857-0)
Supplement: Supplementary file 1 — Supplementary Information [file 41467_2024_45857_MOESM1_ESM.pdf]

Supplementary Information for "Real-time two-axis control of a spin qubit"

Fabrizio Berritta,[*] Torbjørn Rasmussen, Federico Fedele, and Anasua Chatterjee[†]
Center for Quantum Devices, Niels Bohr Institute,
University of Copenhagen, 2100 Copenhagen, Denmark

Jan A. Krzywda and Evert van Nieuwenburg
Lorentz Institute and Leiden Institute of Advanced Computer Science,
Leiden University, P.O. Box 9506, 2300 RA Leiden, The Netherlands

Joost van der Heijden
QDevil, Quantum Machines, 2750 Ballerup, Denmark

Saeed Fallahi
Department of Physics and Astronomy, Purdue University, West Lafayette, Indiana 47907, USA and
Birck Nanotechnology Center, Purdue University, West Lafayette, Indiana 47907, USA

Geoffrey C. Gardner
Birck Nanotechnology Center, Purdue University, West Lafayette, Indiana 47907, USA

Michael J. Manfra
Department of Physics and Astronomy, Purdue University, West Lafayette, Indiana 47907, USA
Birck Nanotechnology Center, Purdue University, West Lafayette, Indiana 47907, USA
Elmore Family School of Electrical and Computer Engineering,
Purdue University, West Lafayette, Indiana 47907, USA and
School of Materials Engineering, Purdue University, West Lafayette, Indiana 47907, USA

Jeroen Danon
Department of Physics, Norwegian University of Science and Technology, NO-7491 Trondheim, Norway

Ferdinand Kuemmeth[‡]
Center for Quantum Devices, Niels Bohr Institute,
University of Copenhagen, 2100 Copenhagen, Denmark and
QDevil, Quantum Machines, 2750 Ballerup, Denmark

(Dated: January 28, 2024)

CONTENTS

Supplementary note 1: Experimental setup 2

Supplementary note 2: Relation between the quality factor and the Bayesian estimation procedure 5
Finite frequency resolution 5
Low quality estimates 6

Supplementary note 3: Extracting exchange energy and Overhauser field gradient from Larmor frequencies 8

Supplementary note 4: Controlled Hadamard rotations 10
Protocol 10
Examples of Hadamard rotations without feedback 11

References 11

SUPPLEMENTARY NOTE 1: EXPERIMENTAL SETUP

Supplementary Supplementary Fig. 1 displays the experimental setup used in this study, which comprises a Triton 200 cryofree dilution refrigerator from Oxford Instruments capable of reaching a base temperature below 30 mK. A superconducting vector magnet is thermally anchored to the 4 K plate and can generate 6 T along the main axis z of the refrigerator and 1 T along x or y. It is used to apply an in-plane magnetic field of $B \approx 200\,\mathrm{mT}$, along the double quantum dot (DQD) axis (see device schematic in Supplementary Fig. 1). An upper bound of the electron temperature is 100 mK, determined by attributing the observed broadening of the interdot (1,1)-(0,2) charge transition to thermal broadening.

The Quantum Machines OPX+ includes real-time classical processing at the core of quantum control with fast analog feedback. It enables on-the-fly pulse manipulation, which is critical for this experiment [1]. The RF carrier frequency, approximately 158 MHz, is attenuated at room temperature by a programmable step attenuator. The RF carrier power incident onto the PCB sample holder corresponds to between -70 and -80 dBm. Before entering the cryostat, the signal is filtered with low-pass and high-pass filters, and a DC block reduces heating in the coaxial lines caused by the DC component of the RF signal. The RF carrier is reflected by the surface-mounted tank circuit wirebonded to one ohmic of the sensor dot and is amplified by a cryogenic amplifier at 4 K. The RF carrier is then amplified again at room temperature, filtered to avoid aliasing, and digitized at 1 GS/s. As the signal is AC coupled and does not have a $50\,\Omega$ resistance to ground, a bias tee is used to bias the input amplifier of the OPX+.

The QDAC-II [2] can trigger the OPX+, for example while tuning the device in video-mode using the RF reflectometry measurements taken by the OPX+. All the ohmics of the device are grounded at the QDevil QBox, a breakout box where low-pass filters are installed for all the DC lines for the gate voltages. To reduce noise and interference in the signal chain, a QDevil QFilter-II [2] is installed in the cryostat for the DC lines. Additionally, the setup includes a QDevil QBoard sample holder [2], which is a printed circuit board with surface-mounted tank circuits used for multiplexed RF reflectometry. The bias tees have a measured cut-off frequency of $\approx 300\,\mathrm{Hz}$, whereas the RC filters have a cut-off frequency $> 30\,\mathrm{kHz}$.

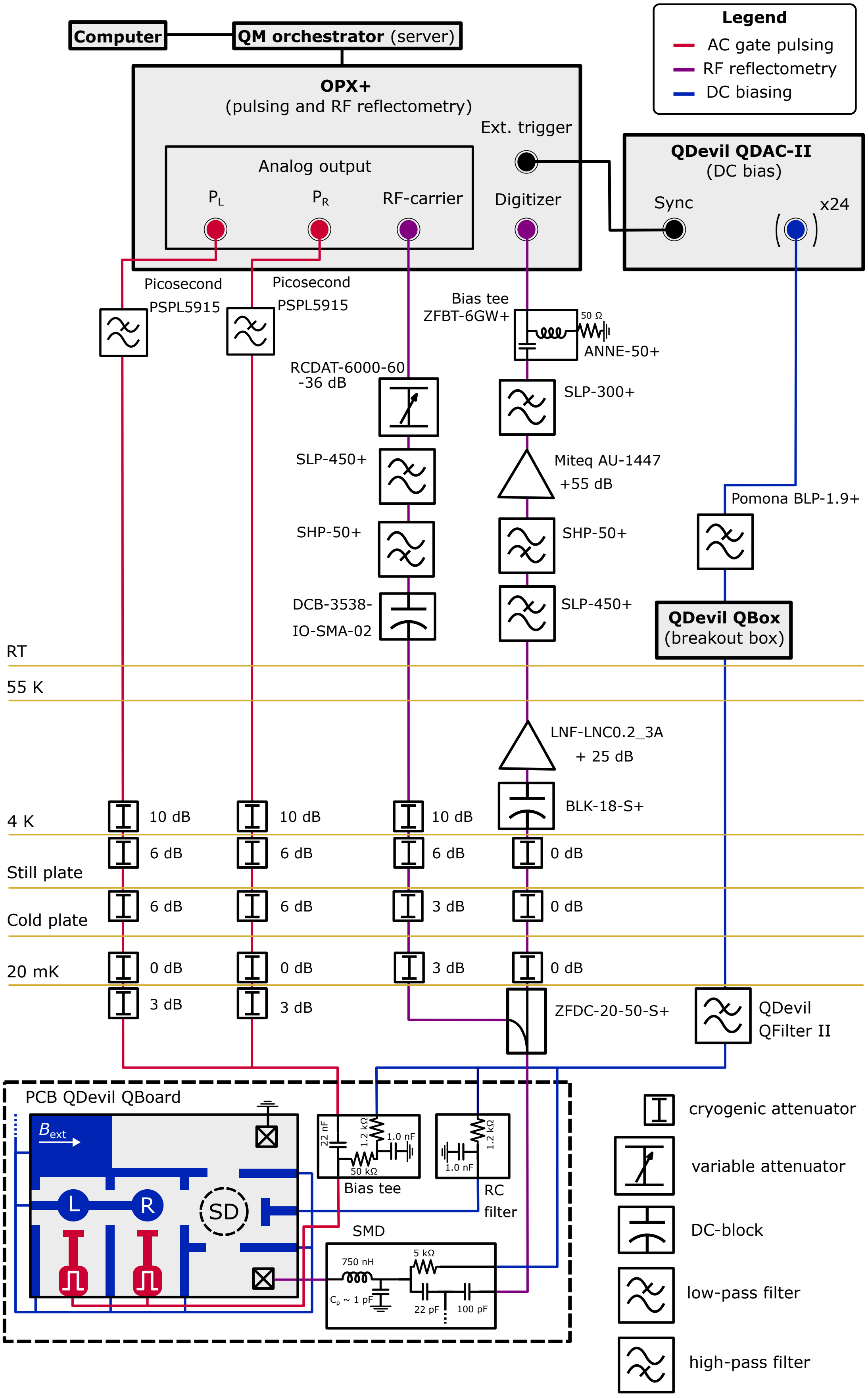

Supplementary Figure 1. See next page for caption.

Supplementary Figure 1. **Experimental setup (previous page).** The cryostat is a Triton 200 dilution refrigerator by Oxford Instruments with a base temperature lower than 30 mK. A Quantum Machines OPX+ is used for RF reflectometry and fast gate control pulses with a bandwidth of less than 1 GHz. The RF carrier frequency used is approximately 158 MHz. Additionally, the setup includes a QDevil QDAC-II for generating low-frequency analog signals. A QDevil QFilter-II is used to suppress noise and interference in the signal chain. Finally, the setup incorporates a QDevil QBoard PCB sample holder with surface-mount tank circuits for multiplexed RF reflectometry. Only one of the four qubits of the chip is activated for this experiment.

SUPPLEMENTARY NOTE 2: RELATION BETWEEN THE QUALITY FACTOR AND THE BAYESIAN ESTIMATION PROCEDURE

In this section we show that most of the oscillation decay, i.e., the finite quality factor of coherent rotations in Fig. 2 in the main text, can be explained by the estimation errors. For each estimation procedure we represent the probability distribution of Ω on a regular grid, and update the weights based on the measurement outcome. To convert the final probability distribution to an estimation of the frequency we use the average:

$$\langle\Omega\rangle = \sum_n p(\Omega[n])\Omega[n] \tag{1}$$

where $\Omega[n]$ is the frequency and $p(\Omega[n])$ is the corresponding probability. When the estimated frequency is used to adjust the time for coherent rotations $t = \phi/(2\pi\langle\Omega\rangle)$ [in the main text the qubit rotation angle $\theta \equiv \phi/(2\pi)$], any estimation error $\delta\Omega$ will result in a random phase evolution $\delta\phi = 2\pi\delta\Omega\, t$ that contributes to a decrease in a quality factor.

Finite frequency resolution

The first source of errors can be associated with the finite resolution of the probability distribution. While too high a resolution would significantly slow down an on-the-fly estimation scheme, too low a resolution would introduce an estimation error, the scale of which can be related to a step size $\delta\Omega \approx (\Omega[n+1] - \Omega[n])/2$.

To optimize the frequency resolution used in the Bayesian estimation, we perform different measurements for both Ω_L and Ω_H. Supplementary Supplementary Fig. 2a shows the averaged traces after performing controlled Overhauser-driven rotations whenever $\langle\Omega_\mathrm{L}\rangle > 30\,\mathrm{MHz}$, as described in Fig. 2 of the main text. Increasing the frequency resolution from 10 MHz to 0.3 MHz (with frequency span from 10 MHz to 70 MHz) results in an increased number of visible oscillations. However, considering the required computational time by the FPGA and no appreciable improvements above 0.5 MHz, we choose a resolution of 0.5 MHz throughout this work, resulting in $\approx 5\,\mu\mathrm{s}$ per qubit cycle to update the estimate on the FPGA.
We also test the frequency resolution of Ω_H in a similar way by performing exchange-driven controlled rotations, as

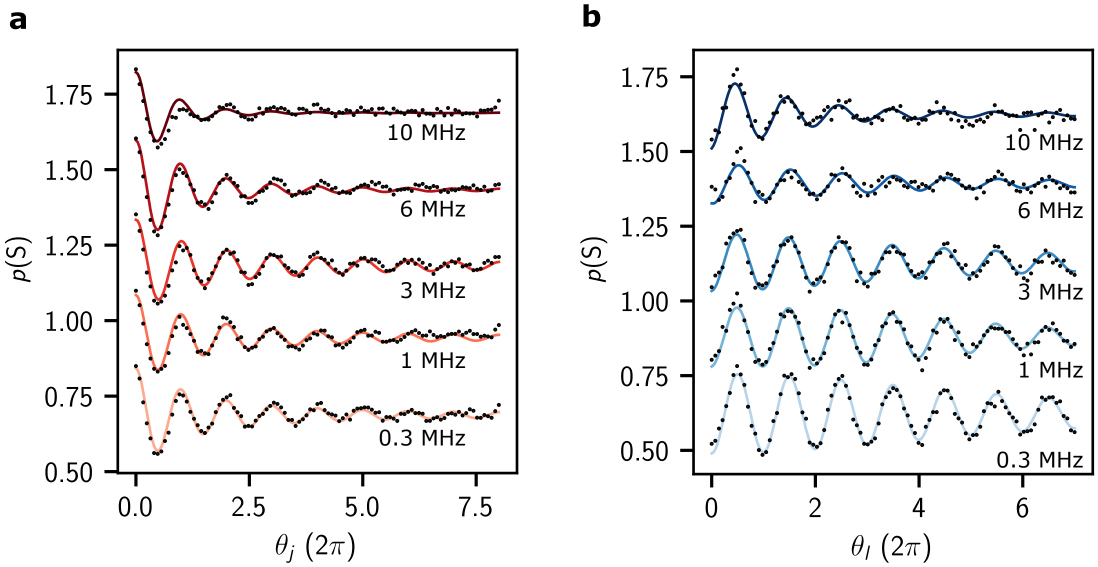

Supplementary Figure 2. **Frequency resolution of Bayesian estimation. a** Each trace shows averaged Overhauser-driven controlled rotations as Fig. 2d in the main text. For each trace we used a different frequency resolution for the probability distribution when estimating Ω_L, as indicated. The y-axis of each curve is offset for clarity. **b** Each trace shows exchange-driven controlled rotations as shown in Fig. 4c in the main text. For each trace we used a different frequency resolution for the probability distribution when estimating Ω_H, keeping fixed the frequency resolution of Ω_L at 1 MHz. The y-axis of each curve is offset for clarity.

shown in Fig. 4 of the main text, and estimating Ω_{H} between 40 MHz and 90 MHz. In this case, we also set the resolution to 0.5 MHz to keep the estimation cycle on the few µs scale.

We support the above by a simple model of uncertainty, in which for each realization of the experiment we draw a random error $\delta\Omega \approx \mathcal{N}(0, \sigma^2_{\delta\Omega})$. For simplicity we assume the initialization and measurement axis are perpendicular to the rotation axis, such that the probability of measuring the initial state reads:

$$P_{\mathrm{S}}(\tau) = \frac{1}{2} + \frac{1}{2}\left\langle \cos(2\pi[\Omega + \delta\Omega]\tau)\right\rangle_{\delta\Omega} = \frac{1}{2} + \frac{\cos(2\pi\,\Omega\tau)}{2}W(\tau), \tag{2}$$

where $W(\tau)$ is the attenuating function related to classical averaging over repetitions of $\delta\Omega$, which we denoted as $\langle \dots \rangle_{\delta\Omega}$. For simplicity we assume the errors are normally distributed with characteristic width $\sigma_{\delta\Omega}$, for which $W(\tau) = \exp\left(-2\pi^2\sigma^2_{\delta\Omega}\tau^2\right)$. For the relevant case of stabilized rotations, which are obtained by adjusting evolution time $\tau = 2\pi\Omega/\phi$, we have

$$P_{\mathrm{S}}(\phi) = \frac{1}{2} + \frac{1}{2}\cos(\phi)\exp\left\{-\frac{1}{2}\left(\frac{\sigma_{\delta\Omega}}{\Omega}\right)^2\phi^2\right\} \equiv \frac{1}{2} + \frac{1}{2}\cos(\phi)\exp\left\{-\left(\frac{\phi}{\phi_2^*}\right)^2\right\}, \tag{3}$$

where in analogy to dephasing time T_2^* we defined dephasing angle ϕ_2^*, which measures the angle of rotation at which the amplitude falls as 1/e. For a constant estimation error it depends on the estimated frequency, since:

$$\phi_2^* = \sqrt{2}\,\frac{\Omega}{\sigma_{\delta\Omega}}, \tag{4}$$

and can be related to a Q-factor via the equation:

$$Q = \frac{\phi_2^*}{2\pi} = \frac{\Omega}{\sqrt{2}\pi\sigma_{\delta\Omega}}. \tag{5}$$

Using the above, we estimate that an resolution-related error of $\sigma_{\delta\Omega} \approx 0.5\,\mathrm{MHz}$ at $\Omega = 20\,\mathrm{MHz}$ (the smallest estimated frequency) should allow for at least $Q \geq 9$. As this number is larger than any measured Q, we conclude the finite resolution of the estimation is not the main factor responsible for observed amplitude decay.

Low quality estimates

Having confirmed sufficiently high resolution (0.5 MHz), another source of errors is associated with low-quality estimates resulting from multi-modal or not sufficiently narrow probability distributions. As a measure of estimation quality we take the variance of the final distribution, defined as:

$$\sigma^2_{\mathrm{est}} = \mathrm{Var}(\Omega) \equiv \sum_n \left(\Omega[n] - \langle\Omega\rangle\right)^2 p(\Omega[n]) \tag{6}$$

To show that low-quality estimates play an important role in the loss of oscillation amplitude we post-process the measured data based on σ_{est}. We reject the repetitions with a probability distribution with calculated variance of $\sigma_{\mathrm{est}} > \sigma_{\mathrm{est,max}}$, and we average over the remaining traces. We highlight that in principle, such or an even more sophisticated procedure of quality assessment could also be performed on-the-fly, for instance as a part of the same feedback loop and without the need of measuring the low-quality oscillations.

We test this approach on the experimental data used to generate Fig. 2d in the main text. In Supplementary Fig. 3a, we plot the obtained probability distributions with $\langle\Omega_L\rangle \geq 50\,\mathrm{MHz}$. For each distribution we compute its variance, whose histogram over all the distribution is plotted in Supplementary Fig. 3b. By imposing an upper bound for the variance $\sigma_{\mathrm{est,max}}$, we can now compute an average such as shown in the bottom panel of Fig. 2d in the main text, but now using only a selection of the best estimates.

In Supplementary Fig. 4b we plot the ratio of used data $\tilde{N}$ versus total repetition $N = 1450$, as a function of $\sigma_{\mathrm{est,max}}$. For each value of $\sigma_{\mathrm{est,max}}$ we plot in Supplementary Fig. 4a the change in the averaged singlet probability $P_{\mathrm{S}}(\theta_j)$ of stabilized oscillations, as compared to the trace shown in the main text. In particular, we focus on two different bounds of tolerated variance $\sigma_{\mathrm{est,max}} = 0.8\,\mathrm{MHz}$ and $2.7\,\mathrm{MHz}$, that correspond to rejecting 80% and 50% of the worst estimations, respectively. We mark those values of $\sigma_{\mathrm{est,max}}$ using dashed lines in both panels. Finally, in Fig 4c we compare the coherent oscillations obtained using the 20% (green) and 50% (yellow) best estimates against the unfiltered result from Fig. 2d (black). We see a significant improvement in the oscillation amplitude, which suggests that the observed decay may be associated with poor performance of the estimation scheme.

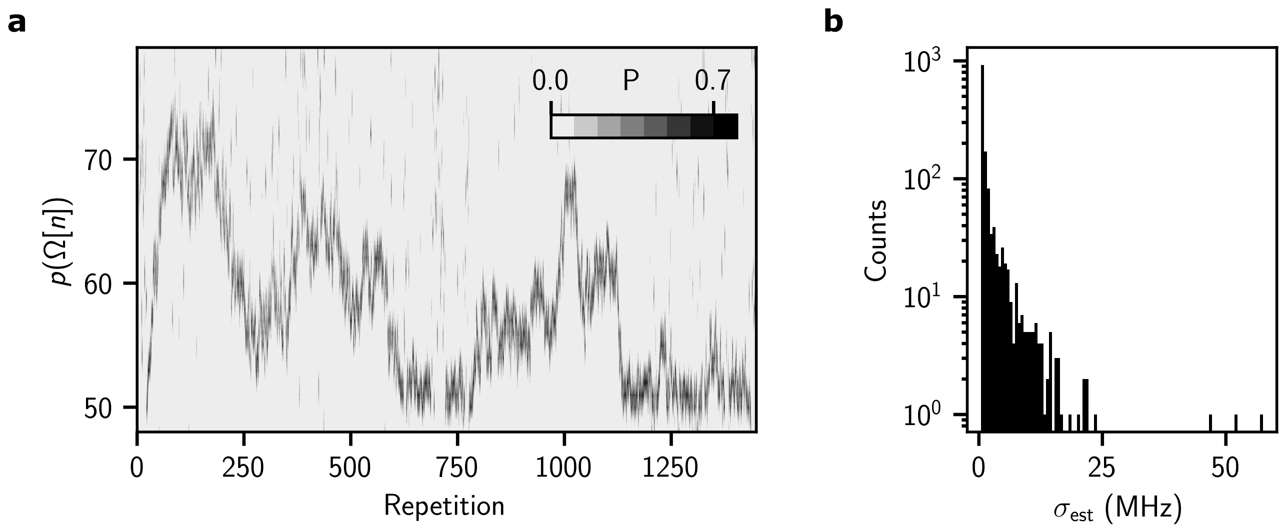

Supplementary Figure 3. **Standard deviation of Bayesian estimations.** **a** Probability distributions used to estimate frequencies $\langle \Omega_L \rangle > 50\,\mathrm{MHz}$. **b** Histogram of corresponding variances σ_{est} of the probability distributions $p(\Omega[n])$.

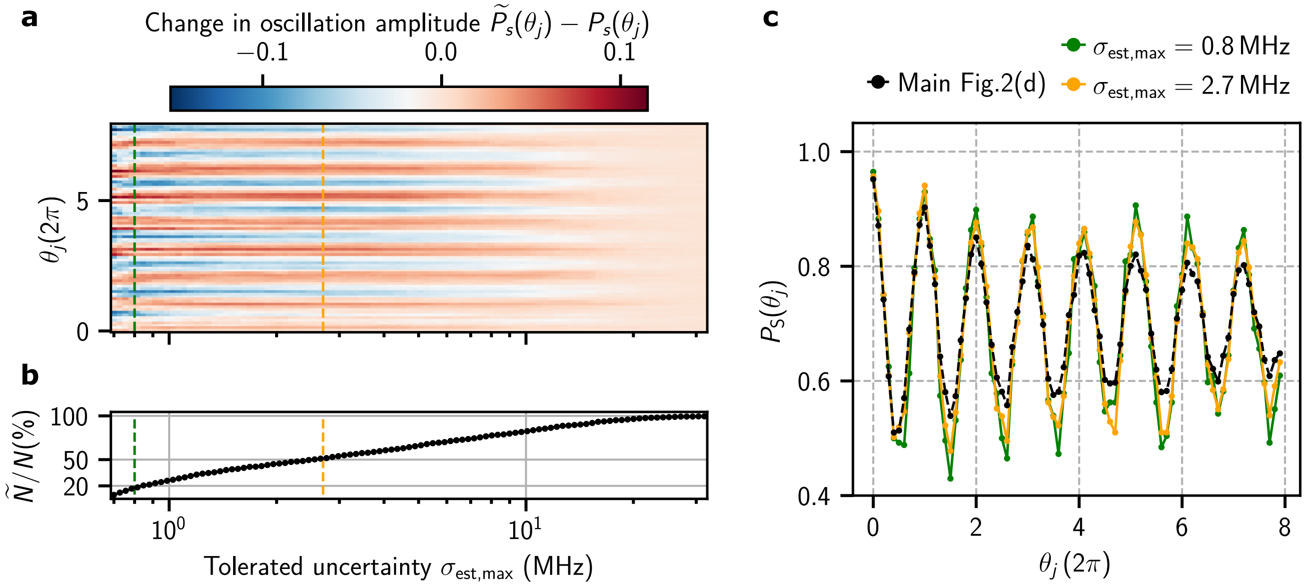

Supplementary Figure 4. **Improvement of the visibility of coherent oscillations by rejecting low-quality estimates.** **a** Change in oscillation amplitude after averaging only the best $\tilde{N}$ estimates out of $N = 1450$ as a function of bound estimation uncertainty $\sigma_{\mathrm{est,max}}$. **b** The fraction of used estimations $\tilde{N}/N$ as a function of bound estimation uncertainty $\sigma_{\mathrm{est,max}}$. By dashed lines we mark $\sigma_{\mathrm{est,max}} = 0.7\,\mathrm{MHz}$ (green), and $2.7\,\mathrm{MHz}$ (yellow), that correspond to rejecting 80% and 50% of repetitions respectively. **c** The resulting averaged oscillations in comparison to unfiltered data (black) from Fig. 2d in the main text.

SUPPLEMENTARY NOTE 3: EXTRACTING EXCHANGE ENERGY AND OVERHAUSER FIELD GRADIENT FROM LARMOR FREQUENCIES

In this section, we seek to extract time-dependent knowledge about the exchange energy, $J(t)$, and the Overhauser field gradient, $\Delta B_z(t)$, from the $\Omega_\mathrm{L}(t)$ data and $\Omega_\mathrm{H}(t)$ data shown in Fig. 3(c,d). To achieve this, we assume $\Omega_\mathrm{L}(t) \equiv \sqrt{\Delta B_z^2(t) + J_\mathrm{res}^2(t)}$ and $\Omega_\mathrm{H}(t) \equiv \sqrt{\Delta B_z^2(t) + J^2(\varepsilon_\mathrm{H}, t)}$, and combine this with statistical methods as the problem is not analytically solvable: at each time t, we have two known quantities [$\Omega_\mathrm{L}(t)$ and $\Omega_\mathrm{H}(t)$] and three unknown ones [$\Delta B_z^2(t)$, $J_\mathrm{res}^2(t)$, and $J^2(\varepsilon_\mathrm{H}, t)$].

As $\Omega_\mathrm{H}(t)$ is probed only when $20\,\mathrm{MHz} < \langle\Omega_\mathrm{L}(t)\rangle < 40\,\mathrm{MHz}$, we downsample $\langle\Omega_\mathrm{L}(t)\rangle$ to the same number of points as we have for $\langle\Omega_\mathrm{H}(t)\rangle$, by choosing for each value of $\langle\Omega_\mathrm{H}(t)\rangle$ the one of $\langle\Omega_\mathrm{L}(t)\rangle$ that is closest in time. We then remove any outliers in the data by considering a window of size $w = 15$ around each data point and reject any data point that is more than the standard deviation of its 14 neighboring data points away from the average of those points. In such a case, we replace the rejected data point with that average. We then finally apply a running average with a window size of $w = 5$ to smoothen the data and remove high-frequency noise. In Supplementary Fig. 5a we show the resulting $\langle\Omega_\mathrm{L,H}(t)\rangle$, cf. Fig. 3(c,d) in the main text.

We assume the fluctuations of $|\Delta B_z|$ to dominate on this time scale, and thus we square Ω_L and Ω_H and determine the shift κ_opt^2 that results in the best overlap of $\langle\Omega_\mathrm{L}(t)\rangle$ and $\sqrt{\langle\Omega_\mathrm{H}(t)\rangle^2 - \kappa^2}$, using a least squares method. The result is plotted in Supplementary Fig. 5b, with $\kappa_\mathrm{opt} \approx 36\,\mathrm{MHz}$, suggesting that on average $J(\varepsilon_\mathrm{H})^2 \approx J_\mathrm{res}^2 + \kappa_\mathrm{opt}^2$. The clear correlation between the two traces confirms the assumption that the fluctuations of $|\Delta B_z|$ dominate over the fluctuations of J. The slightly worse overlap seen in some regions, for instance at about 1 s, are possibly due to fluctuations of J_res and $J(\varepsilon_\mathrm{H})$.

Nevertheless, in order to extract direct knowledge about $\Delta B_z(t)$ we still need to determine $J_\mathrm{res}(t)$ [or $J(\varepsilon_\mathrm{H}, t)$] independently. To obtain an average value of $J_\mathrm{res}(t)$, we combine together longer measurements of $\langle\Omega_\mathrm{L}(t)\rangle$ and $\langle\Omega_\mathrm{H}(t)\rangle$ taken at different times at the same tuning and cooldown over $\approx 36\,\mathrm{s}$ and $\approx 6\,\mathrm{s}$, respectively. We then consider histograms of the measured frequencies without filtering [we show in Supplementary Fig. 5c the one obtained

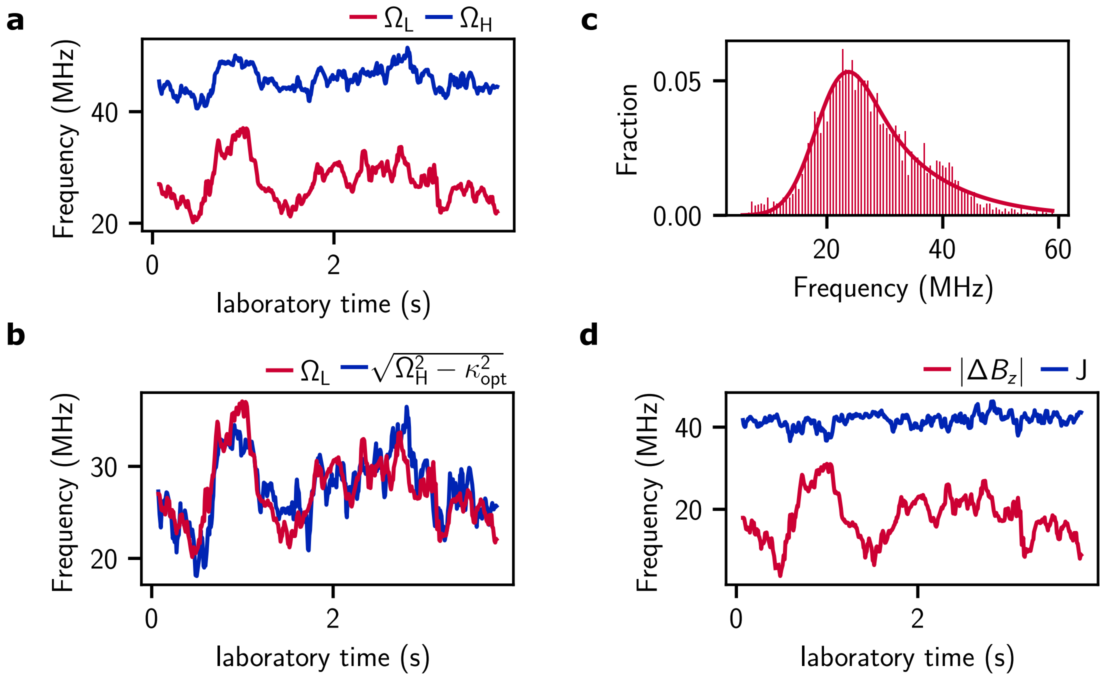

Supplementary Figure 5. **Extracting fluctuations of the exchange energy and Overhauser field gradient from two different estimated Larmor frequencies.** **a** Fluctuations of the Larmor frequencies Ω_L and Ω_H after removal of estimation outliers (see text). **b** The estimated Larmor frequency $\langle\Omega_\mathrm{L}(t)\rangle$ and the shifted frequency $\sqrt{\langle\Omega_\mathrm{H}(t)\rangle^2 - \kappa_\mathrm{opt}^2}$, based on the same data as shown in Fig. 3 of the main text, where κ_opt provides the best overlap using a least-squares error. **c** Histogram of a $\approx 36\,\mathrm{s}$ long measurement of $\langle\Omega_L\rangle$. From a fit to equation (7) (red solid line) the average $J_\mathrm{res} \approx 20.2\,\mathrm{MHz}$ is found. **d** $|\Delta B_z(t)|$ and $J(\varepsilon_\mathrm{H}, t)$ extracted from the Larmor frequencies $\Omega_\mathrm{L}(t)$ and $\Omega_\mathrm{H}(t)$ of main Fig. 3, assuming constant $J_\mathrm{res} = 20.2\,\mathrm{MHz}$.

for $\langle\Omega_\mathrm{L}\rangle$], which we fit to the distribution function

$$\mathcal{P}(\Omega) = \int_{-\Omega}^{\Omega} dJ \frac{1}{\pi\sigma_J\sigma_B} \frac{\Omega}{\sqrt{\Omega^2 - J^2}} \exp\left[-\frac{(J-\mu_J)^2}{2\sigma_J^2}\right] \exp\left[-\frac{\Omega^2 - J^2}{2\sigma_B^2}\right], \tag{7}$$

that gives the probability density for $\Omega = \sqrt{J^2 + B^2}$ when J and B are normally distributed variables with means μ_J and zero and variances σ_J^2 and σ_B^2, respectively. The red solid line in Supplementary Fig. 5c shows the least-square fit of the data, yielding $\sigma_J \approx 4.63\,\mathrm{MHz}$, $\sigma_B \approx 22.0\,\mathrm{MHz}$, $\mu_J \approx 20.2\,\mathrm{MHz}$ for the $\approx 36\,\mathrm{s}$ long trace of $\langle\Omega_\mathrm{L}(t)\rangle$.

The results of this fit confirm that $\sigma_B > \sigma_J$, i.e., that the fluctuations in the Overhauser gradient dominate those in J_res. Indeed, first-order detuning fluctuations of the residual exchange interaction are expected to be zero, as the qubit is tuned close to the symmetry point in the $(1,1)$ charge state when measuring Ω_L [3, 4]. To get a rough picture of the time dependence of $|\Delta B_z(t)|$ we thus replace the residual exchange term $J_\mathrm{res}(t)$ by its constant average value μ_J as extracted from the fit and we extract $|\Delta B_z(t)| = \sqrt{\Omega_\mathrm{L}^2(t) - \mu_J^2}$ and $J(\varepsilon_\mathrm{H}, t) = \sqrt{\Omega_\mathrm{H}^2(t) - \Delta B_z^2(t)}$. The result is plotted in Supplementary Fig. 5d.

SUPPLEMENTARY NOTE 4: CONTROLLED HADAMARD ROTATIONS

Protocol

The aim of this protocol is to perform an adaptive Hadamard gate in a singlet-triplet qubit in GaAs by Bayesian estimation of two Larmor frequencies at different detunings. We define the following quantities:

- $\Omega(\varepsilon) \equiv \sqrt{\Delta B_z^2 + J(\varepsilon)^2}$ is the Larmor frequency at a given detuning ε
- $J_{\rm res}$ is the residual exchange deep in (1,1) ($\varepsilon \approx -40$ mV)
- $\Omega_{\rm L} \equiv \sqrt{\Delta B_z^2 + J_{\rm res}^2}$ is the Larmor frequency deep in the (1,1) charge state.
- At the detuning where $J \approx |\Delta B_z|$ (with $|\Delta B_z| \approx [40, 60]$ MHz), we approximate $J(\varepsilon) \approx J(\varepsilon_0) + \alpha\,\Delta\varepsilon$, where α is ≈ 10 MHz/mV and $\varepsilon_0 \approx -16$ mV
- $\Omega_{\rm H} \equiv \sqrt{\Delta B_z^2 + J(\varepsilon_0)^2}$ is the Larmor frequency at the detuning point defined above
- $\varepsilon_{\rm Had}$ is the detuning at which $J = |\Delta B_z|$, and in general $\varepsilon_{\rm Had} \neq \varepsilon_0$ because J fluctuates.

This protocol assumes we have prior knowledge of the residual exchange (assumed constant), an offline model of $J(\varepsilon)$, and $|\Delta B_z|$ does not depend on detuning ε and it fluctuates sufficiently slowly to be considered constant throughout the protocol. Typical values for $J_{\rm res} \approx [10, 20]$ MHz.

1. Estimate $\Omega_{\rm L}$.
2. Calculate $\Delta B_z^2 = \Omega_{\rm L}^2 - J_{\rm res}^2$. If 40 MHz $< |\Delta B_z| <$ 60 MHz, go to the next point. Otherwise repeat 1.
3. Adjust detuning such that $J = |\Delta B_z|$, based on the offline knowledge of $J(\varepsilon) = J(\varepsilon_0) + \alpha\,\Delta\varepsilon$.
4. To learn the prevailing J, perform exchange-based FID around the field found at 3, i.e. where $J = \Delta B_z$, interleaved with $\Omega_{\rm L}(\pi/2)$ pulses for initialization and readout, from which we estimate $\Omega_{\rm H}$ as in Fig. 3 of the main text.
5. Estimate $J^2 = \Omega_{\rm H}^2 - \Delta B_z^2$ and adjust detuning again such that $J = |\Delta B_z|$ to account for fluctuations of J from the offline model.
6. Perform Hadamard with rotation angle calculated based on $|\Delta B_z|$ by directly jumping to $\varepsilon_{\rm Had}$.
7. Back to 1.

Details

1. Estimate $\Omega_{\rm L}$ by 101 single shots linearly spaced between 0 ns and 100 ns.
2. Calculate $\Delta B_z^2 = \Omega_{\rm L}^2 - J_{\rm res}^2 = (\Omega_{\rm L} + J_{\rm res})(\Omega_{\rm L} - J_{\rm res})$.
3. Adjust detuning such that $J = |\Delta B_z|$. In practice this condition is equivalent to $\Omega_{\rm H}^2 = 2(\Omega_{\rm L}^2 - J_{\rm res}^2)$. Since
$$\Omega_{\rm H}^2(\varepsilon) \approx (J(\varepsilon_0) + \alpha\Delta\epsilon)^2 + \Omega_{\rm L}^2 - J_{\rm res}^2, \tag{8}$$
by solving we get the required detuning shift $\Delta\varepsilon^* = (\Delta B_z - J(\varepsilon_0))/\alpha$.
4. Perform FID with interleaved $\Omega_{\rm L}(\pi/2)$ pulses, from which we measure $\Omega_{\rm H,meas}$.
5. Adjust detuning again such that $J = |\Delta B_z|$. We have
$$\Omega_{\rm H,meas}^2(\varepsilon) \approx (J(\varepsilon_0) + \alpha\Delta\epsilon_{\rm meas})^2 + \Omega_{\rm L}^2 - J_{\rm res}^2, \tag{9}$$
where we have assumed $\Omega_{\rm L}^2$, $J_{\rm res}^2$ and $J(\varepsilon_0)$ have not changed. We look for the fluctuation in detuning by looking at $\Omega_{\rm H,meas}^2 - \Omega_{\rm H}^2$ and find
$$\Delta\varepsilon_{\rm meas} \approx \frac{\Omega_{\rm H,meas}^2 - \Omega_{\rm H}^2}{2J(\varepsilon_0)\alpha} + \Delta\varepsilon^*, \tag{10}$$
having neglected second order terms in $\Delta\varepsilon$. So we shift the detuning of the qubit by the amount $\Delta\varepsilon^* - \Delta\varepsilon_{\rm meas}$.
6. Perform Hadamard with rotation angle calculated based on $\Omega_{\rm Had} = \sqrt{2}|\Delta B_z|$ by directly jumping to $\varepsilon_{\rm Had} = \varepsilon_0 + \Delta\varepsilon^* - \Delta\varepsilon_{\rm meas}$.
7. Back to 1.

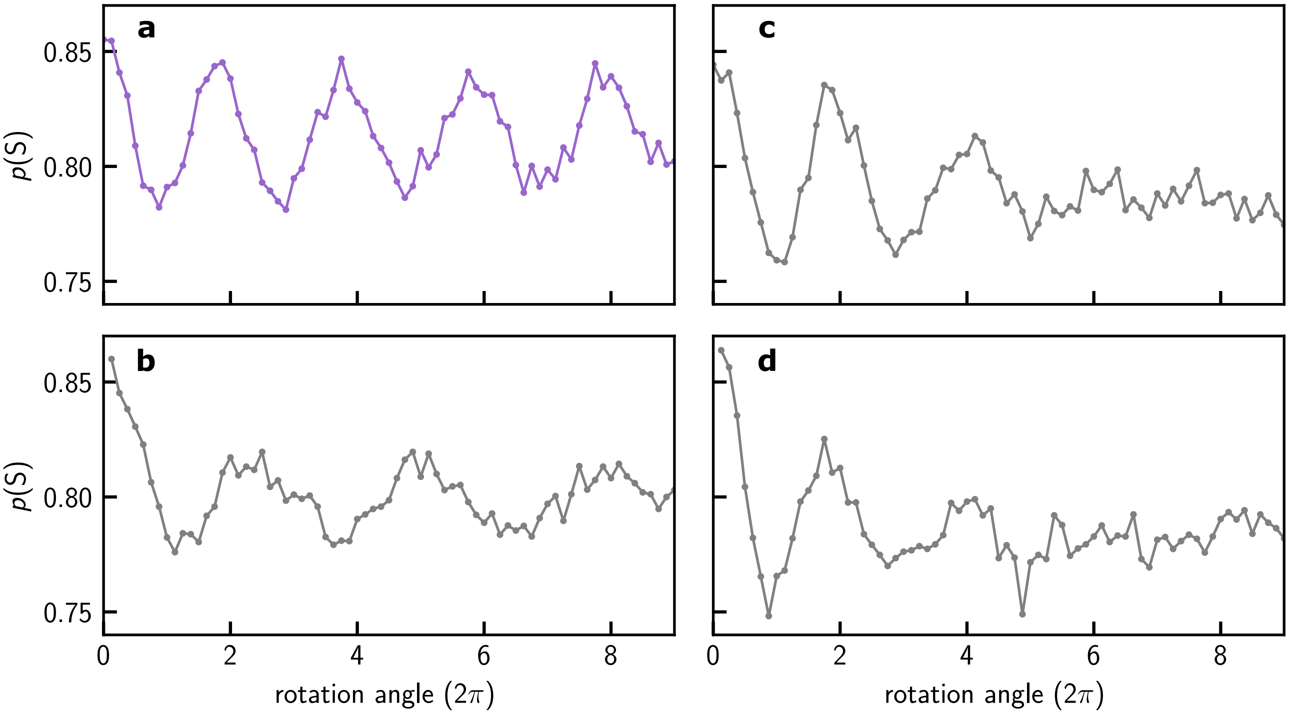

Supplementary Figure 6. **Comparing Hadamard rotations with and without real-time stabilization. a** Measurement of Hadamard rotations with feedback, same data as shown in Fig. 5e of the main text. **b-c-d** Additional measurements of Hadamard rotations without feedback, not shown in the main text.

Examples of Hadamard rotations without feedback

In Supplementary Fig. 6 we compare the quality of Hadamard rotations with feedback (reproduced from Fig. 5e of the main text) with naive Hadamard rotations without feedback, as explained in the main text. In the absence of feedback, the resulting oscillation curves fluctuate randomly in time, even though the cycles of qubit control pulses were nominally identical for panel b, c and d. This is likely due to the Overhauser gradient drifting over time. (All panels were taken within minutes of each other.) For example, data in panel b and d suggests a slight under- and over-rotation relative to the target rotation angles, while in c we have picked a data set that happens to show approximately the correct rotation angles. For all nominally identical uncontrolled Hadamard experiments that we acquired, we observe a quality factor that is much lower compared to the stabilized oscillations in panel a.

* fabrizio.berritta@nbi.ku.dk
† anasua.chatterjee@nbi.ku.dk
‡ kuemmeth@nbi.dk
[1] Quantum Machines, www.quantum-machines.co.
[2] QDevil, www.qdevil.com.
[3] Martins, F. *et al.* Noise suppression using symmetric exchange gates in spin qubits. *Phys. Rev. Lett.* **116**, 116801 (2016).
[4] Reed, M. D. *et al.* Reduced sensitivity to charge noise in semiconductor spin qubits via symmetric operation. *Phys. Rev. Lett.* **116**, 110402 (2016).
